# Fatal outcome in a patient under immunosuppressant therapy infected with human T-lymphotropic virus type 1 (HTLV-1), cytomegalovirus (CMV) and *Strongyloides stercoralis*: a case report

**DOI:** 10.1186/s12879-020-05195-0

**Published:** 2020-07-02

**Authors:** Chisato Ashida, Koji Kinoshita, Yuji Nozaki, Masanori Funauchi

**Affiliations:** Department of Hematology and Rheumatology, Kindai University School of Medicine, 377-2 Oonohigashi, Osaka-Sayama, Osaka, 589-0014 Japan

**Keywords:** Strongyloidiasis, Immune reconstitution inflammatory syndrome, Cytomegalovirus, Case report

## Abstract

**Background:**

Strongyloidiasis is a gastrointestinal parasitic infection caused by percutaneous infection with *Strongyloides stercoralis*. Digestive symptoms such as diarrhea and abdominal pain are the main manifestation, but serious infections such as septicemia, purulent meningitis, and bacterial pneumonia may occur in individuals harboring human T-lymphotropic virus type 1 (HTLV-1) or who are immunocompromised. Although coinfection with *Strongyloides stercoralis* and HTLV-1 can lead to chronic strongyloidiasis and a disseminated form of the disease, there is a high rate of response to the anthelmintic ivermectin.

**Case presentation:**

We report a case of strongyloidiasis infection syndrome that was difficult to differentiate from immune reconstitution inflammatory syndrome (IRIS) for various reasons. The patient had been treated with the corticosteroids tacrolimus (Tac) and mycophenolate mofetil (MMF) for systemic lupus erythematosus (SLE) with lupus nephritis and pancytopenia. When the steroid was reduced, she developed cytomegalovirus (CMV) enteritis, and her respiratory status rapidly deteriorated immediately after the withdrawal of Tac and MMF. It was difficult to distinguish immune reconstitution inflammatory syndrome from strongyloidiasis infection syndrome because stool cultures were negative and eosinophils were not increased. Bronchoscopy revealed viable *Strongyloides*, leading to a diagnosis of strongyloidiasis infection syndrome, but the patient died despite treatment.

**Conclusions:**

Both corticosteroid therapy and HTLV-1 infection can be associated with a decrease of eosinophils, despite the presence of parasitic infection. In conclusion, even if multiple culture tests are negative, the risk of parasitic infection should be assessed in patients receiving immunosuppressants and steroids even in non-endemic areas.

## Background

Strongyloidiasis is a parasitic infection caused by percutaneous infection with the nematode *Strongyloides stercoralis* from the soil. *Strongyloides* species are widely distributed in the tropics and subtropics, and it is estimated that between 30 and 100 million people are infected with *S. stercoralis* worldwide. Strongyloidiasis usually progresses subclinically or with mild abdominal symptoms, but some patients may develop disseminated strongyloidiasis with sepsis, pneumonia, and meningitis. Patients at high risk of *S. stercoralis* infection and infection include those receiving heavy corticoid therapy (because of organ transplants, cancer or autoimmune diseases), those with HTLV-1 coinfection, and chronic alcoholics. The first-line agent for treatment is ivermectin, and the eradication rate is high when it is administered daily for 2 weeks, after confirmation of negative conversion of the worms in culture.

We present a case of cytomegalovirus enteritis that occurred during immunosuppressive therapy for SLE, followed by strongyloidiasis infection syndrome, which resulted in death despite daily administration of ivermectin.

## Case presentation

The patient was a 66-year-old Japanese woman who had immigrated from Okinawa, the only subtropical region of Japan. She presented with a chief complaint of bleeding tendency and had pancytopenia and serositis. HTLV-1 was positive, but adult T-cell leukemia/lymphoma (ATLL) had not developed. Anti-ds-DNA antibody was positive, and both protein and occult blood were present in urine samples. Renal histopathology showed the LNIV type, and SLE was diagnosed. The patient received immunosuppressive therapy with prednisolone, mycophenolate mofetil (MMF) and tacrolimus (Tac), including high-dose intravenous steroid therapy, and her nephritis, hypocomplementemia and pancytopenia improved.

Two months after the start of immunosuppressive therapy, she developed nausea and abdominal pain, and 1 month later she visited our hospital. On admission, she also had severe leg edema and diffuse abdominal tenderness. Laboratory tests showed thrombocytopenia, elevated levels of C-reactive protein, procalcitonin, and alkaline phosphatase, and no eosinophilia. Cytomegalovirus (CMV) pp. 65 antigen was markedly elevated and stool culture tests was negative. Her nephritis had not worsened and pancytopenia had not recurred. Computed tomography (CT) showed thickening of the walls of the ascending colon, colon, and small intestine, an increase in the density of the surrounding adipose tissue, and a fine nodule in the middle lobe of the right lung. Lower gastrointestinal endoscopy demonstrated redness, edema, and erosion from the cecum to the descending colon, but multiple stool culture tests using simple direct smears and the Harada-Mori filter paper culture method were negative.

On the basis of these findings, the patient was diagnosed as having cytomegalovirus enteritis. MMF and Tac were discontinued, ganciclovir was administered, and she was managed by fasting. Despite a temporary improvement in her abdominal symptoms, they became aggravated again. Her respiratory condition then worsened 8 days after admission, necessitating intubation. Because bacterial pneumonia and pneumocystis pneumonia were considered as differential diagnoses, broad-spectrum antibiotics and antifungals were administered, but her respiratory status did not improve, and CT demonstrated diffuse infiltrative shadows and exacerbations.

**Fig. 1:**
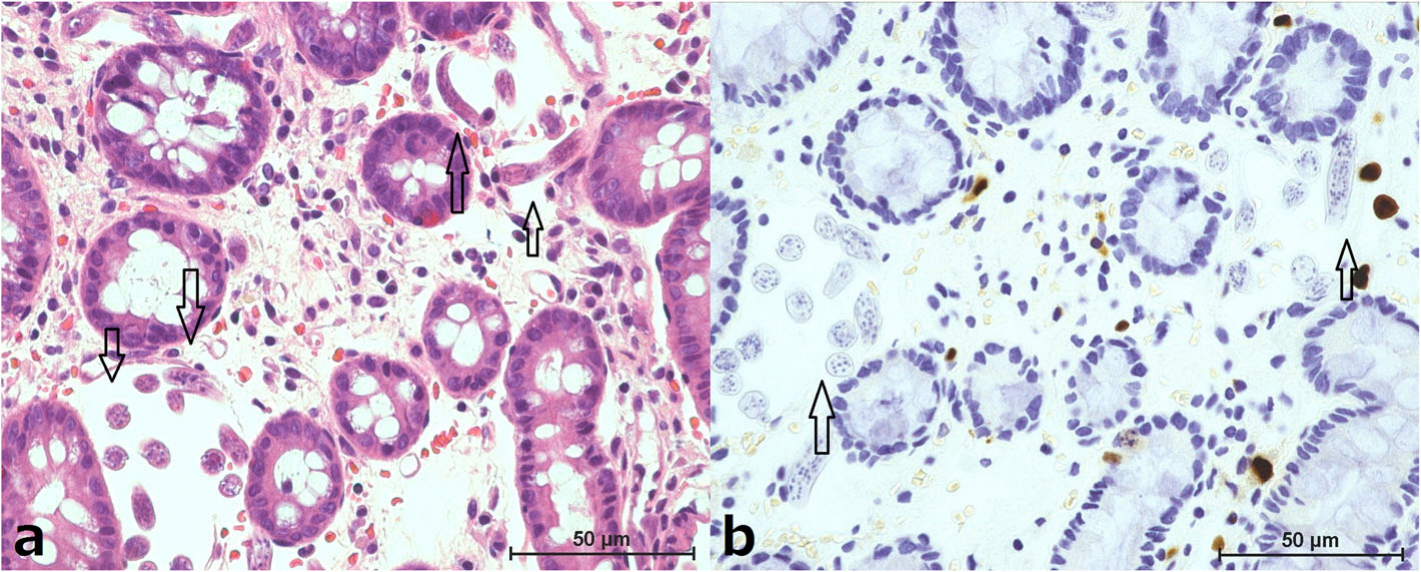
A paracentesis histopathology specimen of the cecum. a: HE stain (× 800 magnification) b: CMV stain (× 800 magnification). Some worms and CMV-positive cells are evident

**Fig. 2:**
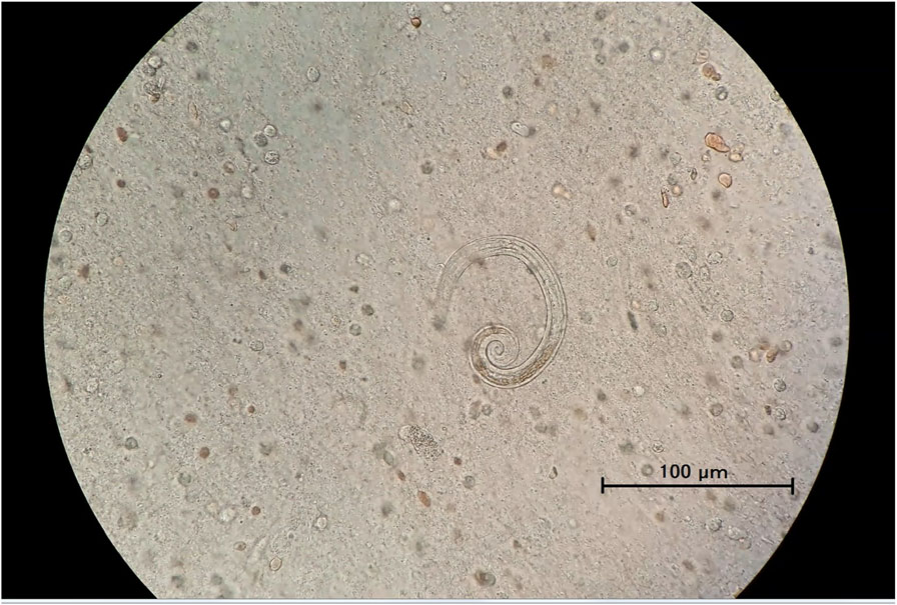
Photomicrograph of *S. stercoralis* (live rhabditoid larva) detected in the bronchoalveolar lavage fluid (× 400 magnification)

A paracentesis histopathology specimen of the cecum demonstrated CMV-positive cells and *S. stercoralis* worms (Fig. 1). Although repeated stool and sputum examinations revealed no eggs or worms, viable *S. stercoralis* worms was detected in bronchoalveolar lavage fluid collected by bronchoscopy (Fig. 2 and Additional file 1). Based on these findings, the patient was diagnosed as having strongyloidiasis infection syndrome and received 200 μg/kg ivermectin daily starting at 15 days after admission. Though 400 mg albendazole daily was additionally administered after 31 days of hospitalization, the patient developed consciousness disturbance thereafter. Cerebrospinal fluid examination showed an increase in the protein level and cell count, but no *S. stercoralis* worms. The patient died 38 days after admission.

## Discussion and conclusion

*S. stercoralis* is a gastrointestinal parasitic nematode. The worm is widely distributed in tropical and subtropical regions of Africa, Asia, and South America, and in Japan it is endemic to Okinawa and Amami. In Japan, there is an overlap between areas where *Strongyloides* and HTLV-1 are endemic, and it is known that the rate of *S. stercoralis* infection is twice as high in HTLV-1-infected than in non-infected individuals [1].

Affected individuals may be asymptomatic or experience gastrointestinal symptoms such as abdominal pain, bloating, and diarrhea. In addition, in patients with HTLV-1 coinfection or those receiving steroid, large numbers of intestinal bacteria may enter the blood with the worms, resulting in serious infections such as pulmonary mycoplasmosis, pyogenic meningitis and bacterial pneumonia [2, 3].

Ivermectin is the first choice for treatment of strongyloidiasis infection and usually administered twice at 2-week intervals. If immunodeficiency or pulmonary lesions are present, ivermectin is administered daily until fecal or sputum specimens are clear of the organism. The effectiveness of ivermectin for eradication of *S. stercoralis* is high (98%), and there are no serious side effects. However, the deworming rate is known to be low in the presence of HTLV-1 superinfection [4].

A diagnosis of strongyloidiasis should be suspected in individuals with clinical signs and symptoms and eosinophilia. Definitive diagnosis of strongyloidiasis is usually based on the detection of larvae in stools, but in cases of chronic infection this may be insufficiently sensitive as the amount of intestinal parasites is often very low. Although there have been many case reports of disseminated strongyloidiasis and strongyloidiasis hyperinfection, the organism was detected by stool culture, unlike the present case [5].

One of the difficulties in this case was the difficulty in differentiating between immune reconstitution inflammatory syndrome (IRIS) and strongyloidiosis infection, as frequent stool and sputum cultures were negative and no eosinophilia was evident, despite worsening of respiratory symptoms immediately after discontinuation of immunosuppressive drugs and up to the time of bronchoscopy.

Given the expected presence of erosions and redness in the duodenum in view of the long-term presence of strongyloidiosis [3] and the fact that lower gastrointestinal endoscopy had revealed erosions extending from the cecum to the inferior colon, we believe that strongyloidiasis had spread throughout the patient’s intestine. Despite having developed such severe strongyloidiosis, multiple stool culture tests continued to be negative. Multiple tests for the presence of worm bodies, such as the direct smear method and the Harada-Mori filter paper culture test, were performed over several days. However, since several studies have shown that the filter paper culture method is less sensitive than the agar plate culture method [6], the approach we employed may have partly explained why stool culture results remained negative.

The present patient’s respiratory condition deteriorated rapidly after withdrawal of the immunosuppressant, and IRIS was considered as a differential diagnosis. IRIS is an inflammatory reaction caused by reconstitution of a patient’s immune system upon functional recovery of monocytes, macrophages and NK cells, or through an increase in CD4+ cell count, which triggers an excessive immune response to any pathogenic microorganisms present in the body. IRIS can be secondary to human immunodeficiency virus (HIV), drug-induced hypersensitivity syndrome, autoimmune diseases, pregnancy, and internal malignancies. Disorders consistent with IRIS include tuberculosis, cytomegalovirus infection, herpes zoster, and pneumocystis pneumonia. IRIS in non-HIV occurs upon reduction and withdrawal of immunosuppressants, such as prednisone or tumor necrosis factor-alpha inhibitors [7]. In the present case, only the immunosuppressive agent was withdrawn after development of enteritis, and the steroid was not reduced. However, we concluded that involvement of IRIS could not be completely ruled out because the cytomegalovirus enteritis occurred at the time of steroid reduction.

Although *S. stercoralis* has been described as an IRIS-associated pathogen, it has been underreported [8]. The majority of these IRISs have occurred after ART therapy for HIV in patients with HIV-related chronic fecal nematode infestation [9, 10], and there are very few reports of patients who have received immunosuppressive therapy for a short period of only 3 months.

Another issue in the present case was to identify the factor that had played the most important role in the fatal outcome [11]. CMV is an important opportunistic pathogen that causes morbidity and mortality in immunocompromised patients, leading to symptoms such as fever, interstitial pneumonia, enteritis, hepatitis, retinitis, and encephalitis. CMV infection can be assessed through a variety of techniques, including serology and histopathology for detection of viral components, among which phosphorylated protein 65 (PP65 antigenemia), which is abundantly present in viral fragments, is detected in peripheral blood leukocytes by indirect immunofluorescence, and for which early monitoring is indicated for patients with SLE and other diseases requiring immunosuppressive therapy [12]. The present patient had been treated with ganciclovir for CMV infection since the time of admission, and her CMV pp65 continued to decline without leukopenia, despite her worsening condition. Although SLE is known to cause impairment of consciousness and renal function, enteritis, and pancytopenia, these features almost always vary in parallel with biomarkers such as antibodies and complement. The patient had no hypocomplementemia or elevated antibody titers after initiation of remission induction therapy for SLE until the time of death. Therefore, we believe that CMV infection and SLE were also unlikely to have been the main cause of death. Although we suspect that strongyloidiosis infection was a major factor involved in the fatal outcome, the patient did not receive any sequential parasitological follow-up in view of the negative results of culture tests, making it difficult to assess the severity of strongyloidiosis over time. For the same reason, it is difficult to conclude whether the patient would have responded to ivermectin or albendazole. The fatal outcome may have resulted from the ineffectiveness of ivermectin on a background of HTLV1 positivity and renal impairment, or some other combined interaction, such as the impact on MMF or Tac discontinuation on strongyloidiosis and IRIS.

As parasitic infection in a patient undergoing dose reduction of immunosuppressants in a non-endemic area is of considerable interest, we consider this case to be of medical significance. In addition, being able to present a video of living worms in the alveolar lavage fluid and to pathologically confirm worms with CMV-positive cells are features of considerable interest. In summary, this report has described a fatal case of refractory strongyloidiasis infection secondary to CMV infection during immunosuppressive therapy. Parasitic infections should always be borne in mind when administering immunosuppressants to migrants from tropical or subtropical areas.

## Supplementary information

**Additional file 1.**

## Data Availability

The datasets used and/or analysed during the present study are available from the corresponding author on reasonable request.

## Abbreviations

HTLV-1: human T-cell leukemia virus type 1
IRIS: Immune reconstitution inflammatory syndrome
Tac: Tacrolimus
MMF: Mycophenolate mofetil
SLE: Systemic lupus erythematosus
CMV: Cytomegalovirus
ATLL: Adult t-cell leukemia/lymphoma
CT: Computed tomography
HIV: Human immunodeficiency virus
